# Sleep disturbances and dermatology-related quality of life in children with cutaneous mastocytosis: a patient-centered perspective

**DOI:** 10.1007/s00431-026-07148-2

**Published:** 2026-06-25

**Authors:** Leman Tuba Karakurt, Pınar Yağmur Altınkaynak, Dilek Kacar, Ayşe Cavidan Sonkur, Mustafa Arga

**Affiliations:** Division of Pediatric Allergy and Immunology, Department of Pediatrics, Faculty of Medicine, Istanbul Medeniyet University, Istanbul, Turkey

**Keywords:** Dermatology life quality, Pediatric cutaneous mastocytosis, SCORMA Index, Sleep disturbances, Sleep quality

## Abstract

Pediatric cutaneous mastocytosis is a heterogeneous mast cell disorder that typically presents as cutaneous disease with a benign and self-limiting course. Despite its favorable prognosis, children with cutaneous mastocytosis may experience symptom-related effects on daily functioning that have not been comprehensively characterized. Data on sleep disturbances and QoL in pediatric cutaneous mastocytosis, unfortunately, remain limited. This study aimed to evaluate the relationship between sleep parameters and dermatology-related QoL and assess whether disease severity modifies the association between sleep disturbances and QoL. Thirty-five children with confirmed cutaneous mastocytosis and 50 healthy controls were enrolled. Disease severity was assessed with SCORing MAstocytosis (SCORMA) Index. Sleep characteristics were evaluated with Children’s Sleep Habits Questionnaire (CSHQ), and dermatology-related QoL was assessed with Children’s Dermatology Life Quality Index (CDLQI). Children with cutaneous mastocytosis exhibited significantly higher total CSHQ scores compared with controls (median 47.0 vs 39.0, respectively) (*p* = 0.004), with a markedly higher proportion exceeding the clinical screening threshold for sleep disturbance (85.7% vs 31.4%, respectively) (*p* < 0.001). Patients demonstrated increased bedtime resistance, sleep anxiety, night wakings, parasomnias, and daytime sleepiness. CDLQI impairment was generally mild, with 94.2% of patients reporting no or small impact. CDLQI scores correlated strongly with SCORMA scores (*ρ* = 0.57,* p* < 0.001) but were not significantly associated with overall sleep disturbance.

*Conclusion*: Children with cutaneous mastocytosis frequently exhibit sleep disturbances, whereas the impact on dermatology-related QoL was generally mild in most patients. Notably, sleep problems were highly prevalent even when dermatology-related QoL impact was minimal, indicating that dermatology-specific QoL instruments may not fully capture the sleep-related burden of pediatric cutaneous mastocytosis. These findings support the integration of routine sleep and dermatology-related QoL screening into the comprehensive management of pediatric cutaneous mastocytosis.

**Table:** 

**What is Known:** • *Cutaneous mastocytosis in children generally has a favorable prognosis, but symptoms such as pruritus and flushing may affect daily functioning.* • *Data regarding sleep disturbances and dermatology-related quality of life in pediatric cutaneous mastocytosis are limited.*
**What is New:** • *Children with cutaneous mastocytosis had significantly more sleep disturbances than healthy controls, with most patients exceeding the clinical screening threshold for sleep problems.* • *Sleep disturbances were common even among patients reporting minimal impairment in dermatology-related quality of life, suggesting that dermatology-specific QoL measures may underestimate disease burden.*

**What is Known:**

• *Cutaneous mastocytosis in children generally has a favorable prognosis, but symptoms such as pruritus and flushing may affect daily functioning.*

• *Data regarding sleep disturbances and dermatology-related quality of life in pediatric cutaneous mastocytosis are limited.*

**What is New:**

• *Children with cutaneous mastocytosis had significantly more sleep disturbances than healthy controls, with most patients exceeding the clinical screening threshold for sleep problems.*

• *Sleep disturbances were common even among patients reporting minimal impairment in dermatology-related quality of life, suggesting that dermatology-specific QoL measures may underestimate disease burden.*

## Introduction

Mastocytosis is a heterogeneous clonal disorder characterized by the abnormal proliferation and accumulation of mast cells (MCs) in one or more organs and tissues, with clinical manifestations driven largely by mediator release [1]. Based on the pattern of organ involvement, two principal forms are recognized: cutaneous mastocytosis (CM), in which the skin is the sole affected tissue, and systemic mastocytosis, defined by mast cell infiltrates in extracutaneous organs such as the bone marrow, with or without concomitant cutaneous involvement [1]. Although nearly all affected children present with skin lesions, systematic evaluation for extracutaneous involvement is not routinely performed in the pediatric population; the disease in childhood is therefore generally regarded as cutaneous mastocytosis, or “mastocytosis in the skin” [2]. In childhood, CM generally follows a benign, self-limiting course with spontaneous regression toward adolescence [2, 3]. Despite this favorable prognosis, pediatric CM may cause a substantial disease burden during active phases, with symptoms such as pruritus, flushing, blistering, and an ongoing risk of anaphylaxis impairing daily functioning.

The conspicuous appearance of cutaneous lesions may further predispose affected mastocytosis children to stigmatization, peer rejection, social withdrawal, and school absenteeism, amplifying the psychosocial impact of the disease [4]. Emerging evidence suggests that MC infiltration and mediator activity contribute not only to dermatologic and systemic symptoms, but also to fatigue, anorexia, heightened pain perception, and disturbances in sleep architecture, cumulatively leading to marked impairment in health-related quality-of-life (QoL) [5, 6].

While QoL impairment in adult mastocytosis has been extensively documented, data in the pediatric population remain scarce. No disease-specific QoL instrument has yet been validated for children with mastocytosis [7]; however, dermatology-specific measures have proven valuable in capturing the functional, emotional, and social consequences of cutaneous involvement. Sleep is a critical yet underrecognized component of disease burden in pediatric mastocytosis, potentially both reflecting disease activity and amplifying symptom severity and quality-of-life impairment; therefore, understanding its relationship with disease severity and QoL is essential for patient-centered care.

The present study aimed to characterize the prevalence and patterns of sleep disturbances in children with CM, examine the associations between sleep parameters and both disease-specific and dermatology-related QoL outcomes, and determine whether objective markers of disease severity modify the relationship between sleep disturbances and QoL.

## Materials and methods

### Study design and participants

This prospective, questionnaire-based observational study was conducted in a tertiary pediatric allergy and immunology clinic. A total of 35 children aged 4–12 years with a confirmed diagnosis of cutaneous mastocytosis were consecutively enrolled. Diagnosis was established based on clinical findings supported by histopathological evaluation in accordance with accepted diagnostic criteria. Clinical phenotypes were classified at the time of initial diagnosis as maculopapular cutaneous mastocytosis (MPCM), diffuse cutaneous mastocytosis (DCM), mastocytoma, or other, based on the prevailing morphologic appearance of skin lesions and the extent of cutaneous involvement. Patients presenting with widespread or extensive cutaneous involvement were assigned to the DCM category according to the institutional diagnostic practice at the time of enrollment. The study protocol was reviewed and approved by the Ethics Committee of İstanbul Medeniyet University (No: 2025/0216). Inclusion required confirmed diagnosis and written informed consent from caregivers.

### Data collection and assessment tools

Demographic and clinical data (including age, gender, age at diagnosis, disease duration, and clinical presentation) were collected using a standardized data form. Disease severity, QoL, and sleep characteristics were evaluated using validated instruments as outlined below. Disease severity was assessed using the SCORMA (SCORing MAstocytosis) Index, a composite clinical tool that quantifies the extent and intensity of cutaneous lesions alongside associated symptoms, yielding a numerical severity score reflective of cutaneous disease severity [8]. Health-related QoL was assessed using the 10-item Children’s Dermatology Life Quality Index (CDLQI), with total scores categorized according to established banding criteria as indicating no effect, small, moderate, very large, or extremely large effects on the child’s life [9, 10].

Sleep patterns were assessed using the Children’s Sleep Habits Questionnaire (CSHQ), a widely used caregiver-reported instrument designed to evaluate common sleep behaviors and sleep-related problems in children aged 4 to 12 years. The CSHQ yields a total sleep disturbance score and subscale scores across multiple sleep domains, with higher scores indicating greater sleep problems; a total score > 41 is used as the clinical cutoff [11, 12].

### Exclusion criteria

Participants were excluded if they had an acute illness during the assessment period, comorbid conditions known to affect sleep (e.g., allergic rhinitis, asthma, obstructive sleep apnea, or psychiatric/neurological disorders), recent initiation of sleep-affecting medications (within 4 weeks), or incomplete questionnaires (> 10% missing data). Control subjects with any of these conditions or with a history of chronic or dermatologic disease were also excluded.

### Statistical analysis

Statistical analyses were performed using SPSS version 25.0 (IBM Corp., Armonk, NY, USA). Continuous variables were expressed as mean ± standard deviation and categorical variables as frequencies and percentages. Data normality was assessed using the Kolmogorov–Smirnov test. Group comparisons were performed with Student’s *t*-test or ANOVA for normally distributed variables and Mann–Whitney *U* or Kruskal–Wallis tests for non-normal distributions, while categorical variables were analyzed using chi-square tests. Associations between SCORMA Index, CDLQI scores, and sleep parameters were evaluated using Spearman’s rank correlation. A two-sided *p* < 0.05 was considered statistically significant.

No AI-assisted technology was used to generate scientific content, analyze data, or draw conclusions.

## Results

### Participant characteristics

A total of 35 children with cutaneous mastocytosis and 50 controls were included. The mastocytosis and control groups were comparable with respect to age (median 95.8 vs 97.3 months, respectively; *p* = 0.08) and gender distribution, with similar proportions of males (57.1% vs 58.0%, respectively; *p* = 0.937). Among patients, the median age at diagnosis was 12.0 months (IQR 8.0–21.0). Diffuse cutaneous mastocytosis (DCM) was the most frequent lesion type (48.6%), followed by maculopapular cutaneous mastocytosis (MPCM) (22.9%) and mastocytoma (14.3%) (Table 1).

**Table 1:** Demographics and clinical characteristics of patients and control group

Characteristics	
Gender (male/female) (*n *(%))	
Patients Control	20 (57.1)/15 (42.9) 29 (58.0)/21 (42.0)
Current age (months) (mean (SD ±))	
Patients Control	95.8 (± 40.1) 97.3 (± 36.4)
Diagnosis age (months) (median (IQR))	12.0 (8.0–21.0)
Lesion type (*n *(%))	
DCM MPCM Mastocytoma Other	17 (48.6) 8 (22.9) 5 (14.3) 5 (14.3)

*DCM* diffuse cutaneous mastocytosis, *MPCM* maculopapular cutaneous mastocytosis, *max* maximum, *min* minimum, *SD* standard deviation

### Sleep outcomes: mastocytosis vs controls

Overall sleep disturbance burden was higher in the cutaneous mastocytosis group. The total CSHQ score was significantly increased in patients compared with controls (median 47.0 vs 39.0, respectively) (*p* = 0.004). Consistently, the proportion of participants exceeding the clinical screening threshold (> 41) was markedly higher in the cutaneous mastocytosis group (85.7% vs 31.4%, respectively) (*p* < 0.001) (Table 2).

**Table 2:** The comparison of CSHQ total and subscale scores between patients and controls

Sleep parameters	Patients (*n* = 35)	Controls (*n* = 50)	*p*
CSHQ			
Total score (median (IQR)) > 41 (*n* (%))	47.0 (42.0–56.0) 30 (85.7)	39.0 (35.0–46.0) 16 (31.4)	0.004 < 0.001
Bedtime resistance (median (IQR))	9.0 (7.0–12.0)	7.0 (5.0–8.0)	0.001
Sleep onset delay (median (IQR))	1.0 (1.0–1.0)	1.00 (1.0–1.0)	0.296
Sleep duration (median (IQR))	3.00 (3.0–4.0)	3.00 (3.0–3.0)	0.314
Sleep anxiety (median (IQR))	7.0 (4.0–9.5)	5.0 (4.0–6.0)	0.005
Night waking (median (IQR))	4.0 (3.0–5.0)	3.0 (3.0–3.0)	< 0.001
Parasomnia (median (IQR))	9.0 (7.0–10.5)	8.0 (7.0–9.0)	0.008
Sleep-disordered breathing (median (IQR))	3.0 (3.0–3.5)	3.0 (3.0–4.0)	0.217
Daytime sleepiness (median (IQR))	12.0 (10.0–15.5)	8.0 (7.0–9.0)	< 0.001

CSHQ children’s sleep habits questionnaire, IQR interquartile range

Across subscales, patients demonstrated higher bedtime resistance (*p* = 0.001), higher sleep anxiety (*p* = 0.005), more night wakings (*p* < 0.001), higher parasomnia scores (*p* = 0.008), and higher daytime sleepiness (*p* < 0.001). In contrast, there were no statistically significant between-group differences in sleep onset delay (*p* = 0.296), sleep duration (*p* = 0.314), or sleep-disordered breathing scores (*p* = 0.217) (Table 2).

### Associations between sleep parameters and clinical variables in patients

Clinical parameters were analyzed according to clinical phenotype (DCM vs MPCM), revealing no significant differences in sleep-related outcomes assessed by the CSHQ (Table 3).

**Table 3:** The comparison of clinical parameters by clinical phenotype

Parameters	Clinical phenotype			*p*
	DCM	MPCM	Mastocytoma	
**CSHQ (mean (SD ±))**				
Total sleep score Bedtime resistance Daytime sleepiness	49.3 (± 8.2) 9.8 (± 3.5) 14.5 (± 4.2)	48.5 (± 8.2) 9.7 (± 3.6) 12.5 (± 3.0)	43.4 (± 3.4) 9.2 (± 2.2) 11.0 (± 1.4)	0.093 0.780 0.066
**SCORMA** (mean (SD ±))	36.8 (± 16.8)	31.3 (± 20.7)	18.1 (± 10.0)	0.007
**CDLQI (mean (SD ±))**				
Personal relationships Treatment Symptom and feelings Total	0.1 (± 0.05) 0.1 (± 0.03) 0.4 (± 0.6) 1.3 (± 1.7)	0.5 (± 1.1) 0.3 (± 0.4) 0.6 (± 0.3) 2.5 (± 3.9)	0.1 (± 0.03) 0.1 (± 0.01) 0.4 (± 0.3) 0.7 (± 0.6)	0.017 0.043 0.268 0.310

CDLQI children’s dermatology life quality index, CSHQ children's sleep habits questionnaire, DCM diffuse cutaneous mastocytosis, MPCM maculopapular cutaneous mastocytosis, SCORMA SCORing MAstocytosis index, SD standard deviation

Total sleep scores, bedtime resistance, and daytime sleepiness were comparable between the three phenotypic groups (all *p* > 0.05) (Table 3).

**Fig. 1:**
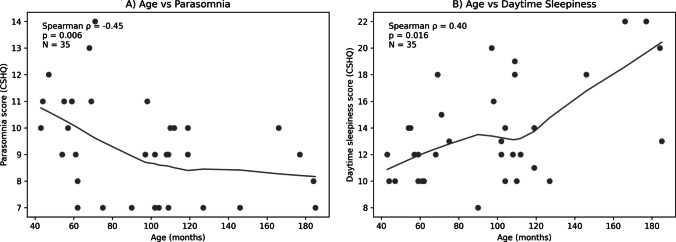
Age-related associations with parasomnia (**A**) and daytime sleepiness (**B**) in children with mastocytosis. Age was negatively correlated with parasomnia and positively correlated with daytime sleepiness

Age was moderately negative correlated with parasomnia scores (*ρ* =  − 0.45, *p* = 0.006) and positively correlated with daytime sleepiness (*ρ* = 0.40, *p* = 0.016) (Fig. 1A and B) (Table 4). No significant associations were observed between age and total CSHQ score (*p* = 0.962) or other subscales (Table 4). Sleep parameters did not differ by gender across the examined domains (all *p* > 0.05) (Table 4). Differences by lesion type were not statistically significant for most sleep outcomes, with borderline signals for daytime sleepiness (*p* = 0.066) and total CSHQ score (*p* = 0.093) (Table 4).

**Table 4:** Associations between sleep parameters and clinical variables in the mastocytosis group

Sleep parameter (CSHQ)	Age correlation^*^ (*ρ*)	Age correlation (*p*)	Gender^§^ (*p*)	Lesion type^∂^ (*p*)
**CSHQ**				
Total sleep score Bedtime resistance Sleep onset delay Sleep duration Sleep anxiety Night awakenings Parasomnias Sleep-disordered breathing Daytime sleepiness	− 0.01 − 0.15 0.07 0.14 − 0.25 − 0.21 − 0.45 − 0.19 0.40	0.962 0.403 0.706 0.410 0.142 0.234 0.006 0.284 0.016	0.726 0.893 0.716 1.000 0.812 0.494 0.413 0.247 0.211	0.093 0.780 0.137 0.437 0.887 0.183 0.240 0.790 0.066
**CDLQI**				
Total score Symptoms and feelings Leisure School or holidays Personal relationships Sleep Treatment	0.02 0.07 − 0.13 − 0.30 − 0.06 0.07 0.00	0.724 0.706 0.461 0.083 0.753 0.684 0.978	0.526 0.257 0.874 0.731 0.137 0.211 0.143	0.309 0.268 0.513 0.643 0.463 0.597 0.643
**SCORMA**	0.34	0.042	0.325	0.006

CDLQI Children's Dermatology Life Quality Index

SCORMA SCORing MAstocytosis Index

CSHQ children’s sleep habits questionnaire

^*^Age correlations were assessed using Spearman’s rank correlation coefficient (*ρ*)

^§^Gender comparisons were performed using the Mann–Whitney *U* test

^∂^Differences across lesion types were evaluated using the Kruskal–Wallis test

### CDLQI in patients

Dermatology-related QoL impairment was generally low. The CDLQI total score was 1.0 (IQR 0–2.5) (Table 5). When categorized by established banding, 62.8% of patients had no effect (0–1), 31.4% had a small effect (2–6), 2.9% had a moderate effect (7–12), and 2.9% had a very large effect (13–18), with no patient scoring in the extremely large effect range (19–30) (Table 5). CDLQI total score was not associated with age and gender, or lesion type (*p* = 0.724, *p* = 0.526, and *p* = 0.309, respectively) (Table 4). Moreover, CDLQI total score was not significantly correlated with overall sleep disturbance (*ρ* = 0.20, *p* = 0.240). Phenotype-specific differences were observed in selected domains of health-related QoL evaluated using the CDLQI. Patients with MPCM demonstrated significantly greater impairment in the personal relationships and treatment domains compared with patients with DCM (*p* = 0.017 and *p* = 0.043, respectively) (Table 3). No statistically significant differences were detected between phenotypes in the symptoms and feelings domain or in the overall CDLQI score (both *p* > 0.05) (Table 3).

**Table 5:** Dermatology-related quality of life and disease severity in children with mastocytosis

**Parameters**	Patients (*n* = 35)
**CDLQI**	
Total score (median (IQR)) Effect (*n* (%)) No (0–1) Small (2–6) Moderate (7–12) Very large (13–18) Extremely large (19–30)	1.0 (0–2.5) 22 (62.8) 11 (31.4) 1 (2.9) 1 (2.9) 0 (0)
**SCORMA**	
Total score (mean (SD ±))	34.4 (18.4)

CDLQI children’s dermatology life quality index, IQR interquartile range, SCORMA SCORing MAstocytosis, SD standard deviation

### Disease severity (SCORMA) and relationships with sleep and QoL

**Fig. 2:**
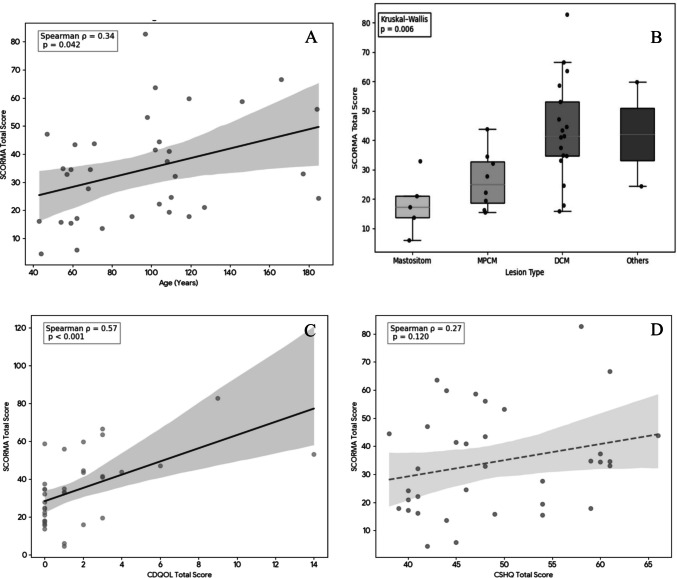
**A** Age was moderately and positively correlated with SCORMA total score (*ρ* = *0.34, p* = *0.042*). **B** A significant difference in disease severity was observed among lesion types (*p* = *0.006)*. **C** Association between CDLQI and disease severity, showing a strong positive correlation (*ρ* = *0.57, p* < *0.001*). **D** No significant association was observed between total CSHQ score and SCORMA total score (*ρ* = *0.27, p* = *0.120*). CDLQI, Children’s Dermatology Life Quality Index; CSHQ, Children’s Sleep Habits Questionnaire; SCORMA, SCORing MAstocytosis

The mean SCORMA total score was 34.4 (SD ± 18.4) (Table 5). SCORMA total score showed a moderately positive correlation with age (*ρ* = 0.34, *p* = 0.042) and differed significantly across lesion types (*p* = 0.006), while no significant difference was observed by gender (*p* = 0.325) (Table 4) (Fig. 2A and B). SCORMA total score was strongly correlated with CDLQI total score (*ρ* = 0.57, *p* < 0.001) (Fig. 2C). In contrast, SCORMA total score was not significantly correlated with CSHQ total score (*ρ* = 0.27, *p* = 0.120) (Fig. 2D). SCORMA scores differed significantly according to clinical phenotype (*p* = 0.007). Patients with DCM (36.8 ± 16.8) exhibited the highest disease severity, followed by MPCM (31.3 ± 20.7), whereas mastocytoma (18.1 ± 10.0) patients had substantially lower SCORMA values (18.1 ± 10.0).

## Discussion

This study highlights that children with cutaneous mastocytosis experience a high prevalence of sleep disturbances, whereas the impact on dermatology-specific QoL was generally limited in most patients. These findings indicate that pediatric cutaneous mastocytosis affects children beyond cutaneous manifestations, particularly through measurable disturbances in sleep, while the dermatology-related QoL impact varies considerably between individuals. By focusing on patient-reported sleep disturbances and QoL, this study highlights under-recognized aspects of disease burden that are not fully captured by conventional clinical assessments and deserve greater attention in routine care.

Sleep disturbances were common in children with mastocytosis [13]. Parents frequently reported prolonged sleep latency, nocturnal awakenings, and poor sleep quality, findings that are biologically plausible given mast cell–mediated disease mechanisms [13, 14]. Pruritus, one of the most prevalent symptoms in pediatric cutaneous mastocytosis, often worsens at night and is likely a key contributor to sleep disruption through histamine and other mast cell–derived mediators affecting both skin symptoms and central sleep regulation [13, 15]. Similar associations between disease activity and impaired sleep have been consistently reported in other mast cell–mediated conditions, such as atopic dermatitis and chronic urticaria, where pruritus severity correlates with fragmented sleep and daytime dysfunction [16, 17]. Our findings align with this literature and suggest a bidirectional relationship in which nocturnal symptoms impair sleep, while sleep deprivation may further exacerbate mast cell activation. From a clinical perspective, these findings suggest that routine screening for sleep disturbances may help identify unmet needs that are not captured by conventional assessments of disease severity or skin-related QoL. Of note, although total CSHQ scores were markedly elevated in patients compared with controls, sleep disturbance did not correlate significantly with SCORMA scores within the patient group. This apparent dissociation may reflect the fact that sleep impairment in pediatric cutaneous mastocytosis is driven by intermittent, mediator-related events such as nocturnal pruritus, flushing, and parental anxiety regarding anaphylaxis, rather than by the cumulative extent of cutaneous involvement captured by SCORMA. In addition, the restricted variability of SCORMA scores in our cohort, together with the relatively narrow age range, may have limited the statistical power to detect such an association. This dissociation also invites a more critical reading of the SCORMA Index itself, which was developed to quantify the extent and intensity of cutaneous lesions and therefore primarily reflects cutaneous disease activity rather than the full multidimensional burden of mastocytosis [8]. Consistent with this, recent consensus criteria in mast cell disorders emphasize that cutaneous and biochemical indices alone do not adequately capture mediator-related symptom severity or QoL impairment, and recommend their combined use with validated patient-reported outcome measures [18]. Interpreted in this light, the strong SCORMA–CDLQI correlation in our cohort likely reflects the convergence of both instruments on the cutaneous dimension of disease, whereas the lack of correlation with CSHQ suggests that sleep disturbance arises from mediator-driven and behavioral mechanisms lying outside the construct captured by SCORMA.

A particularly noteworthy finding of the present study is the dissociation between sleep disturbance and dermatology-related QoL. Whereas 85.7% of patients exceeded the clinical screening threshold for sleep disturbance on the CSHQ, 94.2% of the same patients reported either no effect or only a small effect on the CDLQI, and total CSHQ scores were not significantly associated with total CDLQI scores within the patient group. This pattern indicates that the substantial sleep-related burden experienced by children with cutaneous mastocytosis is not adequately captured by a dermatology-specific QoL instrument. The CDLQI, although well validated and widely used in pediatric dermatology, addresses sleep through a single item (Q9) embedded within a broader assessment of skin-related impact [19]; psychometric analyses have shown that this single item often exhibits low variance and limited contribution to internal consistency, suggesting that it may be insufficient to reflect the multidimensional aspects of pediatric sleep impairment, including bedtime resistance, sleep anxiety, parasomnias, and daytime sleepiness, all of which were prominently affected in our cohort [11, 19]. Similar discordances between objective sleep impairment and dermatology-specific QoL have been reported in pediatric atopic dermatitis, where sleep disturbance constitutes a distinct dimension of disease burden that is incompletely reflected by skin-focused QoL instruments [16, 20, 21]. From a clinical perspective, this finding suggests that reliance on dermatology-specific QoL tools alone may underestimate the overall disease burden in pediatric cutaneous mastocytosis and supports the routine combined use of sleep-specific and dermatology-specific instruments in both clinical follow-up and future research. Methodologically, this dissociation also points to a potential need for disease-specific QoL instruments tailored to pediatric mastocytosis that explicitly incorporate sleep-related dimensions alongside cutaneous and psychosocial domains [22].

Health-related QoL assessment revealed that, in the majority of children with cutaneous mastocytosis, the impact of the disease on dermatology-specific QoL was limited, with most patients falling within the “no effect” or “small effect” CDLQI categories. Compared to the general population, patients demonstrated lower QoL scores, with the greatest impairment observed in the personal relationships and treatment domains of the CDLQI. Beyond these directly measured domains, prior pediatric studies suggest that embarrassment related to visible skin lesions, anxiety regarding unpredictable symptoms, and avoidance of sports or outdoor activities due to fear of symptom exacerbation or anaphylaxis may further contribute to the disease experience, although these specific constructs were not directly assessed in the present study [22, 23]. These findings are consistent with previous pediatric studies reporting emotional distress, social self-consciousness, and peer-related challenges in children with cutaneous mastocytosis [24, 25]. Overall QoL impairment was largely mild, with few patients demonstrating moderate impairment, consistent with prior reports. This contrasts with adult mastocytosis populations, where moderate-to-severe QoL impairment is common, likely reflecting the predominantly cutaneous, self-limited course of childhood disease and its tendency toward spontaneous regression [26]. Although the magnitude of QoL impairment was modest in most patients, a non-negligible minority experienced moderate or very large effects, indicating that QoL screening remains relevant in clinical follow-up and may help identify the subgroup of children for whom the disease meaningfully affects daily life.

An apparently counterintuitive observation is that patients in the MPCM subgroup exhibited greater impairment in the personal relationships and treatment domains than those classified as DCM, despite DCM being conventionally regarded as the more extensive cutaneous phenotype. Several factors may contribute to this finding. First, as discussed below, the classification of phenotypes in our cohort may not have strictly conformed to consensus criteria, and several patients with extensive maculopapular involvement may have been categorized as DCM, which would attenuate the expected severity gradient between the two groups. Second, the visible, polymorphic, and unpredictable distribution of MPCM lesions may exert a disproportionate impact on peer interactions and self-image, as captured by the personal relationships domain of the CDLQI, in school-aged children, irrespective of total body surface involvement. Third, treatment-related burden, including the need for topical therapy, antimediator medication, and avoidance behavior, may be perceived as more intrusive by families of children with persistent maculopapular lesions than by those whose disease manifests as generalized cutaneous thickening that is sometimes managed with a more uniform skincare approach. A further potential contributor is age, since older children are generally more socially aware and more sensitive to the visibility of cutaneous lesions and to treatment-related demands; in our cohort, age was significantly correlated with SCORMA scores and with selected sleep parameters, although it did not correlate with the overall CDLQI score, leaving open the possibility that age-related effects are exerted at the level of specific CDLQI domains rather than at the level of the global QoL score. This residual confounding could not be formally adjusted for given the size of the subgroups and should be regarded as a hypothesis-generating observation warranting confirmation in larger pediatric cohorts.

Our findings suggest that dermatology-related QoL impairment, as captured by the CDLQI, was more closely associated with overall symptom burden, indexed by SCORMA scores, than with clinical phenotype, since QoL did not differ substantially between subtypes. Although direct measures of psychosocial functioning, parental anxiety, and child emotional well-being were not assessed in the present study, prior literature in adult and pediatric mast cell disorders suggests that cosmetic impact, symptom unpredictability, and parental concerns regarding anaphylaxis risk may contribute meaningfully to patient and family experience [27, 28]. Considering these literature-based observations alongside our findings, future studies incorporating validated psychosocial and family-centered measures would be valuable in clarifying the broader impact of pediatric cutaneous mastocytosis on patients and caregivers.

A noteworthy observation in our cohort is the relatively high proportion of patients classified as DCM (48.6%), which exceeds the frequencies reported in larger pediatric cohorts where DCM typically accounts for approximately 2–11% of cases [29]. This discrepancy likely reflects variability in the application of phenotypic criteria, as the boundary between extensive or widespread MPCM and true DCM, defined by generalized cutaneous thickening in the absence of individualized hyperpigmented lesions, can be difficult to delineate in clinical practice. Consistent with this interpretation, mean SCORMA scores in our DCM subgroup (36.8 ± 16.8) were comparable to those of MPCM (31.3 ± 20.7) and lower than the markedly elevated values typically observed in true DCM, suggesting that some patients with extensive MPCM may have been classified within the DCM category. This consideration does not alter the principal findings of our study, which were based on aggregate sleep and QoL outcomes across the entire cohort and on the well-validated SCORMA Index, but it should be borne in mind when interpreting phenotype-specific comparisons. Indeed, this classification ambiguity may also account for the lack of significant differences in CSHQ and overall CDLQI outcomes across the three phenotypic groups, as well as for the unexpected pattern of greater psychosocial impairment in the MPCM subgroup, since true between-phenotype differences would be expected to attenuate when the underlying subgroups are not cleanly delineated.

This study has several strengths, including the use of validated pediatric instruments to assess sleep disturbances in children with mastocytosis and a comprehensive evaluation integrating sleep, disease severity, and dermatology-specific QoL. Recruitment from a specialized pediatric allergy and immunology clinic ensured diagnostic accuracy, and despite the rarity of the disease, the sample size is comparable to prior pediatric studies.

Several limitations should be acknowledged, including the relatively small, single-center sample, which may limit generalizability, and the cross-sectional design, which precludes assessment of causality or longitudinal changes. Additionally, sleep and QoL were assessed using validated questionnaires, which are subject to reporting bias and do not provide objective sleep measures. A further consideration is the absence of a standardized prospective re-evaluation of clinical phenotype using strict consensus criteria; consequently, the boundary between extensive MPCM and DCM may not have been uniformly applied, which should be taken into account when interpreting phenotype-stratified analyses. Finally, constructs such as stigmatization, emotional distress, anxiety, and peer relationships were not directly measured; related statements in the “Discussion” are drawn from the broader literature and should be regarded as contextual rather than as findings of the present cohort.

## Conclusion

Pediatric cutaneous mastocytosis is associated with frequent sleep disturbances, whereas the impact on dermatology-related QoL is generally mild in most patients, with only a small subgroup experiencing moderate or higher levels of impairment. Importantly, the dissociation between high sleep disturbance burden and predominantly preserved dermatology-related QoL indicates that dermatology-specific instruments alone may underestimate the overall impact of the disease, supporting the combined use of sleep-specific and dermatology-specific tools in clinical assessment. These findings support a patient-centered management approach that extends beyond cutaneous symptom control to include routine assessment of sleep quality and dermatology-related QoL. Further research using dedicated psychosocial instruments, as well as the development of disease-specific QoL measures for pediatric mastocytosis that incorporate sleep-related dimensions, would help to characterize the broader impact of the disease on children and families.

## Data Availability

The datasets generated and/or analyzed during the current study are available from the corresponding author on reasonable request.
